# Viologen-Based Covalent Organic Frameworks toward Metal-Free Highly Efficient Photocatalytic Hydrogen Evolution

**DOI:** 10.1021/acsami.2c23233

**Published:** 2023-04-05

**Authors:** Sinem Altınışık, Gizem Yanalak, İmren Hatay Patır, Sermet Koyuncu

**Affiliations:** † Canakkale Onsekiz Mart University, Department of Chemical Engineering, 17100 Çanakkale, Türkiye; ‡ Canakkale Onsekiz Mart University, Department of Energy Resources and Management, 17100 Çanakkale, Türkiye; § Selcuk University, Department of Biochemistry, 42130 Konya, Türkiye; ∥ Selcuk University, Department of Biotechnology, 42130 Konya, Türkiye

**Keywords:** covalent organic framework, viologen, carbazole, hydrogen evolution, metal-free photocatalyst

## Abstract

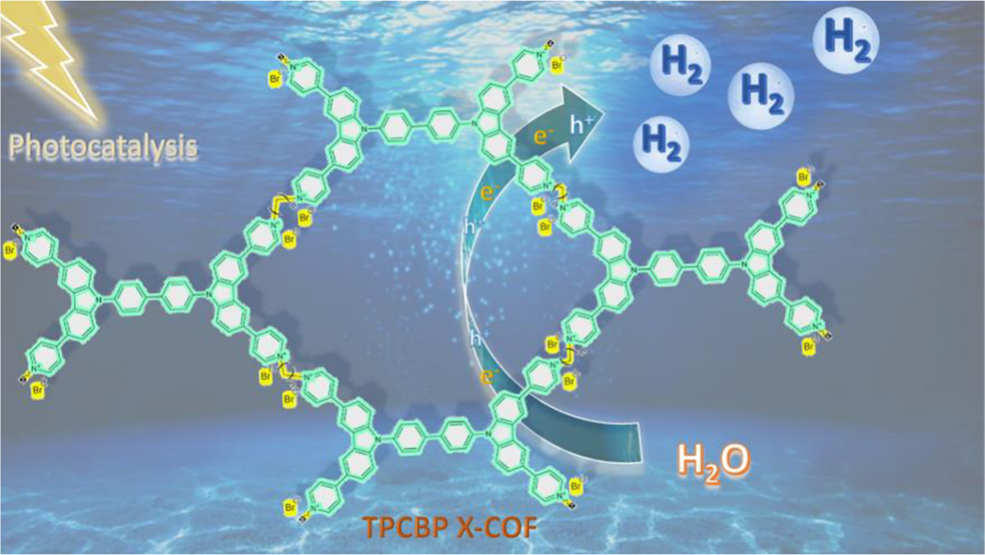

Covalent organic frameworks (COFs) have shown promise in the field of photocatalysts for hydrogen evolution. Many studies have been carried out using various electroactive and photoactive moieties such as triazine, imide, and porphyrin to produce COFs with different geometric structures and units. Electron transfer mediators like viologen and their derivatives can accelerate the transfer of electrons from photosensitizers to active sites. Herein, the combination of a biphenyl-bridged dicarbazole electroactive donor skeleton with a viologen acceptor structure is reported for the photocatalytic hydrogen evolution of novel COF structures with various alkyl linkers {TPCBP X-COF [X = ethyl (E), butyl (B), and hexyl (H)]}. The structures became more flexible and exhibited less crystal behavior as the length of the alkyl chain increased according to scanning and transmission electron microscopy images, X-ray diffraction analyses, and theoretical three-dimensional geometric optimization. In comparison, the H_2_ evolution rate of the TPCBP B-COF (12.276 mmol g^–1^) is 2.15 and 2.38 times higher than those of the TPCBP H-COF (5.697 mmol h^–1^) and TPCBP E-COF (5.165 mmol h^–1^), respectively, under visible light illumination for 8 h. The TPCBP B-COF structure is one of the best-performing catalysts for the corresponding photocatalytic hydrogen evolution in the literature, producing 1.029 mmol g^–1^ h^–1^ with a high apparent quantum efficiency of 79.69% at 470 nm. Our strategy provides new aspects for the design of novel COFs with respect to future metal-free hydrogen evolution by using solar energy conversion.

## Introduction

Photocatalytic water splitting by using semiconductors is one of the hot topics in the field of energy for preventing climate change despite the growing global population and increasing energy demand.^1,2^ In the past few decades, the development of photocatalytic systems that absorb photons for the production of photogenerated electrons and holes for water-splitting reactions has continued unabated.^3^ On the contrary, a photocatalytic hydrogen system presents a significant challenge that requires high-efficiency semiconductors for the conversion of solar energy. The major disadvantages of the photocatalysts are a small surface area, a low crystallinity, a high recombination rate, unfavorable stabilities, and a limited visible light spectrum.^1,4^ Herein, a class of polymers, covalent organic frameworks (COFs), can be suitable for overcoming these obstacles and weaknesses for state-of-the-art photocatalytic hydrogen evolution systems. Since the pioneering work on boron-containing COFs, many papers have described their inherent porosities, stabilities in acidic/basic media, high charge-carrier mobilities, long-range sequential structure, easy functional design, adjustable band gap, etc.^5−7^

On the basis of diquaternized 4,4′-bipyridine moieties, viologens have good reversible redox properties as well as good electron transfer capability. The ability of viologens to undergo reversible redox reactions that give rise to three different oxidation states (MV^2+^, MV^*+^, and MV) is the foundation of many applications, and MV^*+^ is crucial in the photocatalytic process.^8−10^ The neutral state is extremely unfavorable for applications mediated by free radicals, which reduces the efficiency of free radical utilization. Recently, viologen derivatives can also be used in electrochromic devices,^11^ molecular self-assembly,^12^ energy storage,^13^ and catalysts.^14^ However, the application and development of viologens have been severely restricted due to disadvantages such as a wide energy range and a low degree of conjugation. Numerous modification techniques have been developed, such as adding aromatic substituents on either side of the nitrogen atom or adding conjugated groups between two pyridine units and bridging main group elements in the bipyridine moieties.^15−17^ For the development of viologen and related research areas, particularly photocatalysis, maintaining a stable radical state without forming a neutral state during the reducing process became a significant challenge.^18^ However, there have been limited studies on the use of photocatalytic applications via integration of the pyridine-based moiety into the COFs to improve electron transfer capability.^19^

**Figure 1:**
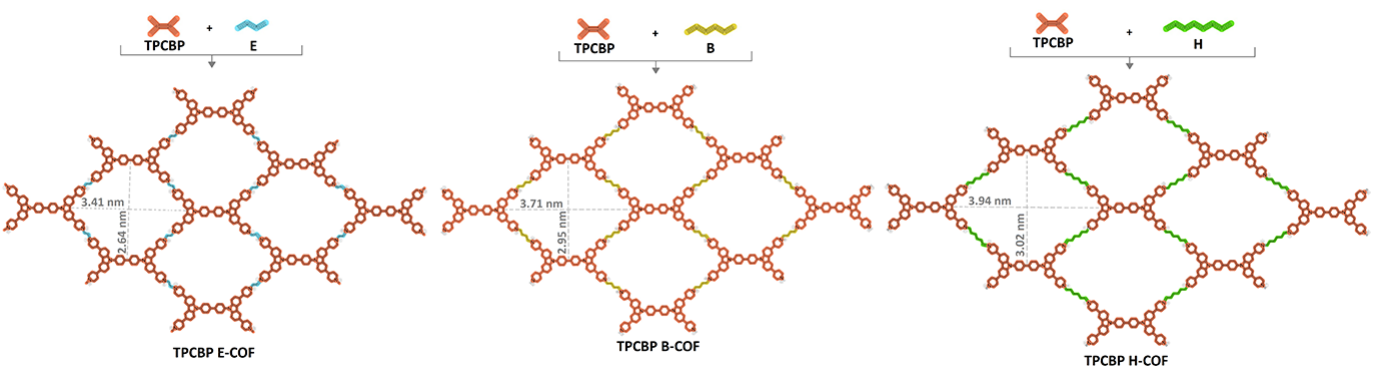
Schematic representation of TPCBP X-COF [X = ethyl (E), butyl (B), and hexyl (H)] structures.

In this work, we focused on the preparation of viologen-based COFs with different length alkyl chain bridges [TPCBP X-COF, where X = ethyl (E), butyl (B), and hexyl (H)], which combine with the carbazole, and their application as metal-free photocatalysts for visible light-driven hydrogen evolution (Figure 1). Because viologens are an efficient electron transfer mediator to pave the way for rapid electron transfer, they can be used in this study for photocatalytic hydrogen evolution by introducing them into the COF structure. In this design, the efficiency of the donor–acceptor interaction between viologen and carbazole at the excited state is an important factor for improving visible light-driven hydrogen production. In addition, the partial separation of electrons and holes is thought to facilitate photocatalytic H_2_ formation by restricting the recombination of photogenerated charge carriers. In the literature, Chen et al. synthesized benzothiadiazole-based COFs via chlorination (Py-ClTP-BT-COF) and fluorination (Py-FTP-BT-COF) for hydrogen evolution, and the HER values were reported as 177.50 and 57.50 μmol h^–1^, respectively.^20^ Gao’s group reported MoS_2_ loaded on a ketoenamine-based TpPa-1-COF catalyst for photocatalytic H_2_ evolution, which was shown to afford HER activity slightly better than that of Pt/TpPa-1-COF.^21^ In another study, Sheng et al. investigated systematically three different groups [X = -H, -(CH_3_)_2_, and -NO_2_] attached to TpPa-COF-X photocatalysts for H_2_ evolution. The HER activities and separation ability of photogenerated charges decreased in the following order: TpPa-COF-(CH_3_)_2_ > TpPa-COF > TpPa-COF-NO_2_.^7^ Considering the importance of the design of COF catalysts in photocatalytic HER systems, several studies have been reported alongside those mentioned above, such as g-C_3_N_4_/CTF-1/Pt (850 μmol h^–1^ g^–1^),^22^ N_2_-COF/Co-1 (782 μmol h^–1^ g^–1^),^23^ A-TEBPY-COF/Pt (98 μmol h^–1^ g^–1^),^24^ TP-BDDA/Pt (324 μmol h^–1^ g^–1^),^25^ BP/CTF (42 μmol h^–1^ g^–1^),^26^ etc. In this work, we suggest a series of TPCBP X-COF [X = ethyl (E), butyl (B), and hexyl (H)] photocatalysts for efficient H_2_ evolution under visible light irradiation. This work revealed that TPCBP X-COFs can not only promote efficient charge separation but also lower the energy barrier for H_2_ production. Among the visible light-driven hydrogen evolution systems with light-harvesting and electron-transferring functions, TPCBP H-COF, TPCBP E-COF, and TPCBP B-COF photocatalysts have hydrogen evolution efficiencies of 296, 489, and 1029 μmol h^–1^ g^–1^, respectively, using only triethanolamine as an electron donor without a cocatalyst. Our findings showed that TPCBP B-COF as a new photocatalyst is one of the materials with the highest hydrogen evolution efficiency and an apparent quantum efficiency (AQE) of 79.69% at 470 nm in the literature. According to our results, when compared with the literature, the photocatalytic HER activities of TPCBP X-COF were shown to be better than that of pristine COF-based photocatalysts, even with a Pt cocatalyst.

## Results and Discussion

### Optical and Electrochemical Properties

**Figure 2:**
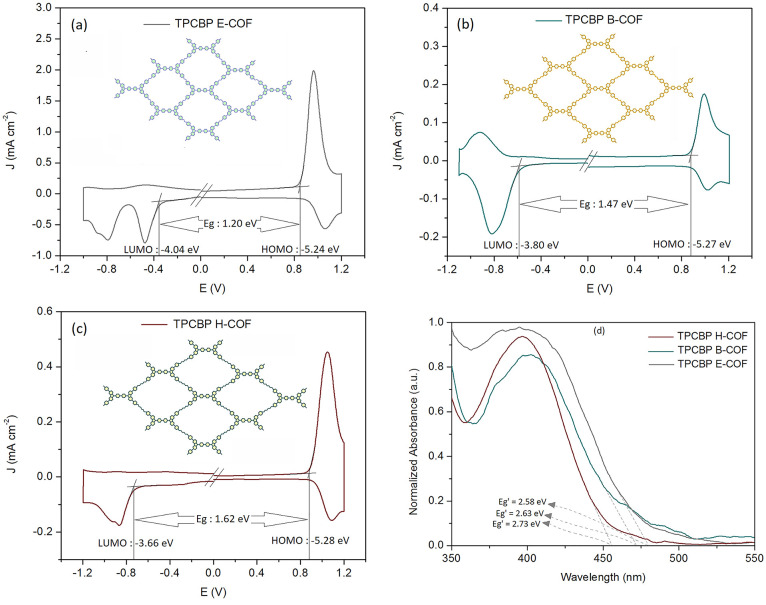
DPV curves of (a) TPCBP E-COF, (b) TPCBP B-COF, and (c) TPCBP H-COF in a 0.1 M TBAPF_6_/ACN electrolyte solution at a scan rate of 100 mV s^–1^, with Ag wire. (d) UV–vis absorption spectra of TPCBP X-COF films on a glass surface.

The synthetic procedure for TPCBP X-COF structures is described in the Supporting Information (Figure S1). TPCBP X-COFs were structurally and thermally characterized by Fourier transform infrared spectroscopy (FT-IR) and thermogravimetric analysis, respectively (Figures S2 and S3). After the synthesis of TPCBP X-COFs, the soluble fraction was separated by Soxhlet extraction with methanol and used for electrochemical and optical characterization. Differential pulse voltammetry (DPV), which is a more efficient method for determining the onset potential of low-solubility materials, was used in electrochemical characterization in addition to cyclic voltammetry (CV) (Figure 2 and Figure S4). The carbazolium radical cation and dication moieties of the repeating CBP structures are responsible for the two-step oxidation peaks that were seen in the anodic scan between ∼1.00 and ∼1.80 V (vs Ag/AgCl) in the CV measurement. In addition, the reduction redox behavior observed between −0.4 and 1.4 V in the cathodic scanning of TPCBP X-COFs indicates that viologens in the structures undergo multiple reduction steps forming a radical cation (MV^+^) and a neutral species (MV^0^). Herein, the potentials of reduction and oxidation of TPCBP E-COF are lower than those of TPCBP B-COF and TPCBP H-COF due to the shorter distance between the carbazole and viologen electroactive moieties and the stronger interaction in the cage. According to these results, the HOMO–LUMO band gap values calculated using DPV depended on the increase in the length of the alkyl bridge, which were found to be 1.20, 1.47, and 1.62 eV, respectively (Figure 2a–c). On the contrary, a similar effect was observed in the solid phase thin film absorption spectrum. Because the interaction between electroactive molecules is stronger in TPCBP E-COF than in other molecules, the COF structure absorbs a regime that is broader than that of others in the ultraviolet–visible (UV–vis) spectrum (Figure 2d). In addition, UV–vis spectra of the soluble fractions of TPCBP X-COFs were recorded in DMF and the formation of a low-energy band centered at 380 nm was observed with a bathochromic shift of ∼40 nm according to the solid phase (Figure S5a). In the photoluminescence (PL) spectra of TPCBP X-COF, bright green emission with a center at ∼550 nm was seen by excitation of all compounds from the lowest-energy band (Figure S5b). From the onsets of the thin film absorption spectrum, the optical band gap values were calculated to be 2.58, 2.63, and 2.73 eV, respectively. The difference between the calculated *E*_g_ and *E*_g_′ of approximately 1.1–1.4 eV shows that the bipolar TPCBP X-COFs containing pyridinium acceptor and carbazole donor units do not interact with each other in the neutral state (Table S1).^27,28^

### Theoretical Calculations

**Figure 3:**
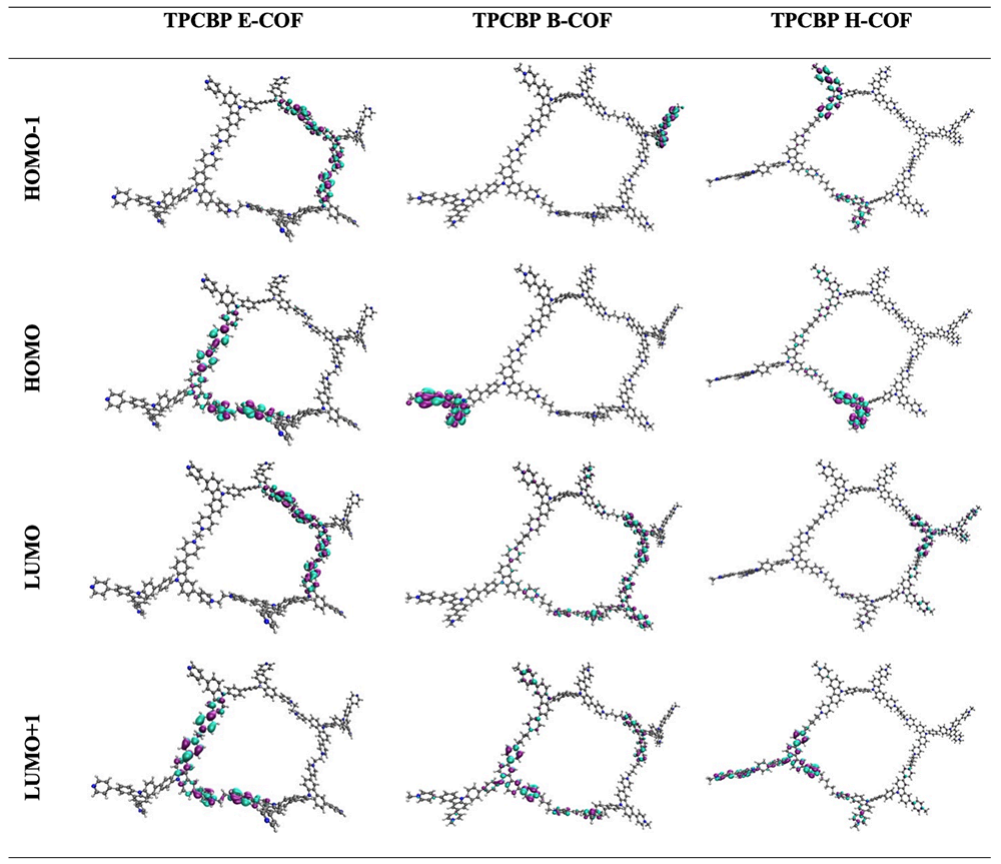
Theoretical HOMO–LUMO charge distribution of TPCBP X-COF structures at the B3LYP/6-31G level.

The bipolar charge separations were also supported by density functional theory (DFT) calculations (Figure 3). According to the DFT results, the charges were located over all conjugated electroactive TPCBP structures at the HOMO and dispersed into the ring at the LUMO. Because the donor–acceptor TPCBP electroactive structures containing carbazole and viologen moieties are closer to each other in TPCBP E-COF, the charge distribution at the HOMO is delocalized over the entire π-system, while the charges are located more specifically on the carbazole donor moiety of the TPCBP B-COF and TPCBP H-COF structures. The charges of the TPCBP B-COF and TPCBP H-COF structures at the LUMO accumulate more on the pyridinium acceptor center throughout the entire ring than for TPCBP E-COF due to the same effect.

### Surface Characterization of TPCBP X-COFs

**Figure 4:**
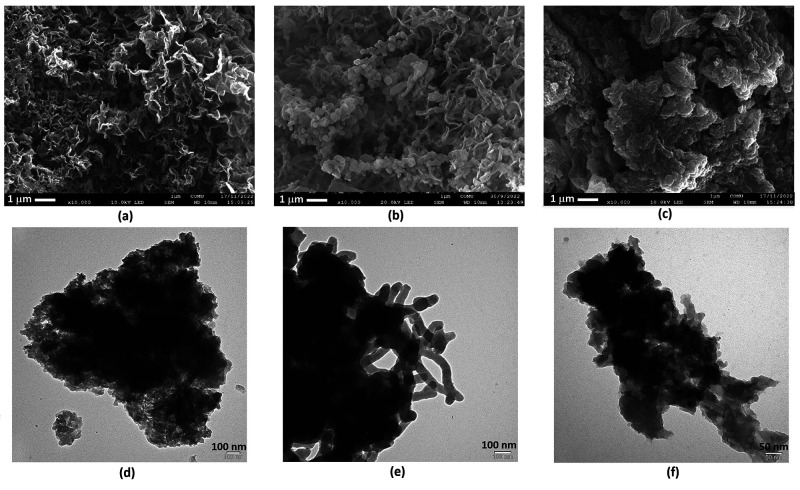
(a–c) SEM images of TPCBP X-COFs and (d–f) TEM images of TPCBP X-COFs dispersed in an ethanol solution (X = ethyl, butyl, and hexyl, respectively).

**Figure 5:**
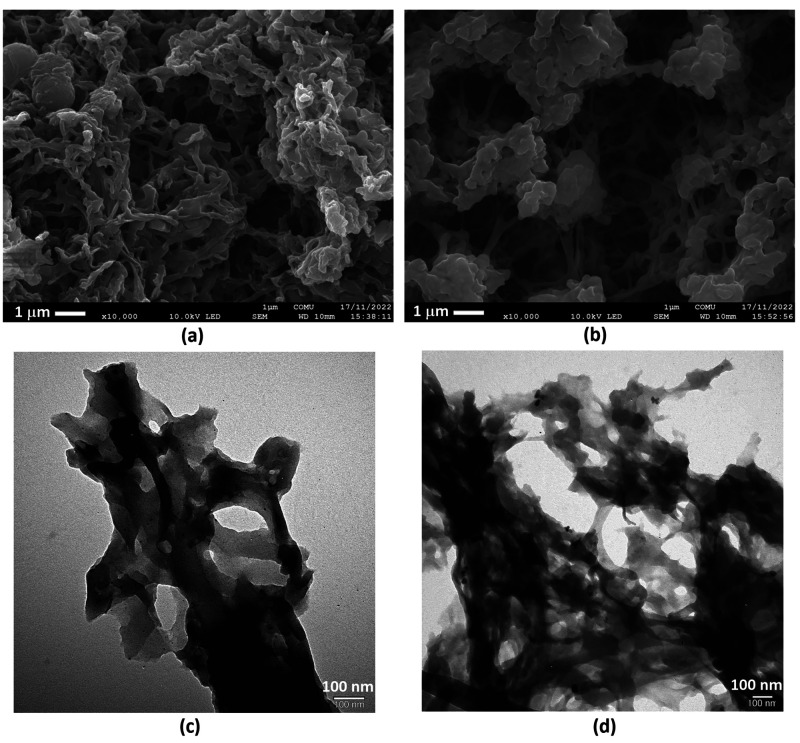
(a and b) SEM and (c and d) TEM images of TPCBP B-COF after visible light illumination for 8 h and a photocatalytic stability test, respectively.

Scanning electron microscopy (SEM) and transmission electron microscopy (TEM) measurements were used to investigate the morphology of the insoluble TPCBP X-COF powders (Figure 4a–f). In addition, the morphological deformations of TPCBP B-COF that occurred as a result of the photocatalytic test were also monitored with SEM and TEM (Figure 5a–d). Due to its shorter bridge length, TPCBP E-COF has been found to have a more crystalline structure with a leaf-like, highly porous surface morphology. As the length of the linker increases, the degree of crystalline character decreases upon addition of a flexible alkyl bridge, and it is observed that aggregation occurs when these leaf-shaped fibers come together. This is also supported by geometric optimization and X-ray diffraction (XRD) pattern as a result of an increased level of bending with elongation of alkyl bridges (Figures S6 and S7).

After the photocatalytic process, the branched particles of TPCBP B-COF with the highest hydrogen formation reaction efficiency join to form an expanded lattice morphology (Figure 5a,b). One can clearly see that the mesoporous holes formed after the photocatalytic stability test are larger than the size of the powder remaining after visible light illumination for 8 h. This result shows that photocatalytic hydrogen evolution takes place via pyridinium bromine salts, and the hollow structure is formed with the depletion of bromine counterions during the reaction. The SEM-EDX results clearly show that the bromine counterions in the hollow structure observed following the photocatalytic hydrogen evolution process were considerably reduced (Figures S8 and S9). Finally, after photocatalytic stability tests, the yellow powder of the TPCBP X-COFs turned gray.

**Figure 6:**
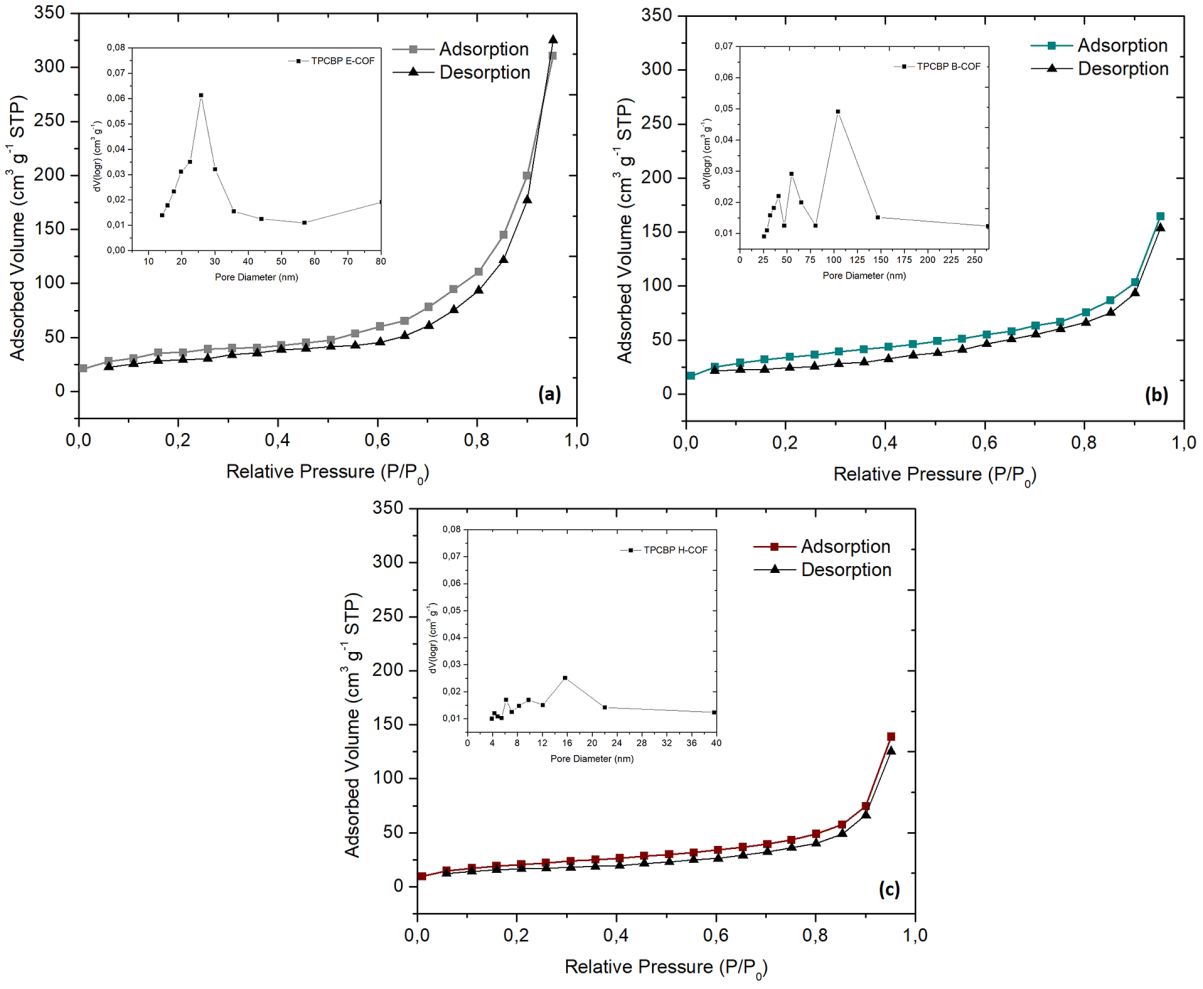
Nitrogen adsorption isotherms of TPCBP X-COF structures.

The specific surface area and pore size distribution of the TPCBP X-COFs were investigated by N_2_ adsorption/desorption measurements at 77 K (Figure 6a–c). There was no drastic initial increase in N_2_ adsorption in the samples in the zone of extremely low pressure, and the structure had macro- and mesoporous surface layers rather than microporous ones. The BET surface area of TPCBP E-COF was calculated as 132.21 m^2^ g^–1^, much larger than those of other COF structures (Table 1). As shown in the BJH plot (insets of Figure 6a–c), the pore distribution of the TPCBP X-COF structures varied at different pore sizes depending on the alkyl chain bridge and agrees with the TEM results. It has been observed that the pores in the TPCBP E-COF structure are ∼20 nm; with alkyl bridge elongation in the TPCBP B-COF structure, the pores expand to ∼60 nm. On the contrary, in the TPCBP H-COF structure, the pores were closed with the elongated alkyl bridge, and pores of ∼15 nm were observed.

**Table 1:** Surface Areas of TPCBP X-COF Structures

molecule	BET surface area (m^2^ g^–1^)	BJH adsorption surface area (m^2^ g^–1^)
TPCBP E-COF	132.21	220.08
TPCBP B-COF	109.82	66.02
TPCBP H-COF	74.01	52.26

### Photocatalytic Hydrogen Evolution Performance of TPCBP X-COFs

**Figure 7:**
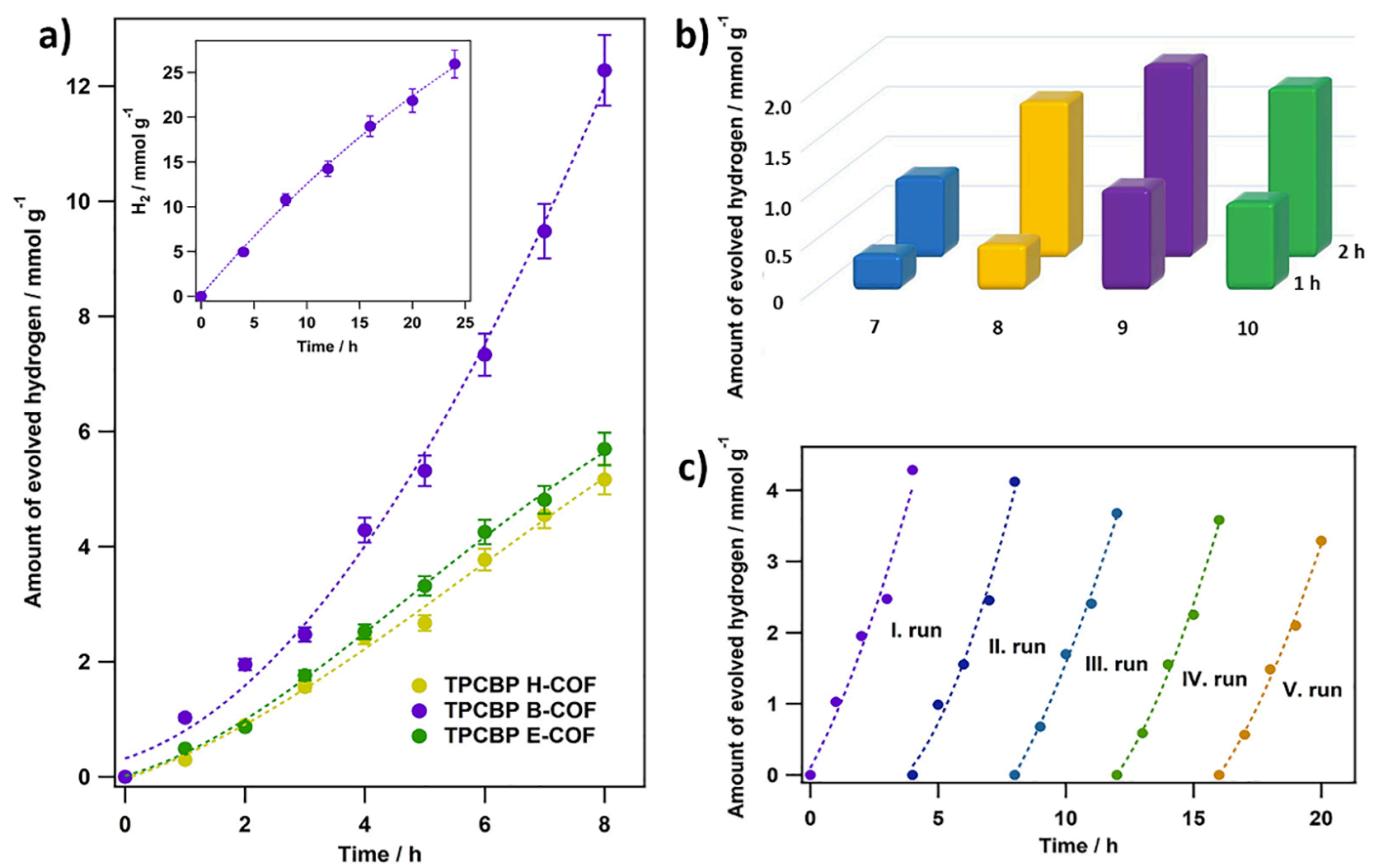
(a) Photocatalytic H_2_ evolution activities of TPCBP X-COFs (X = ethyl, butyl, and hexyl), (b) pH effect of TEOA, and (c) photocatalytic stability test of TPCBP X-COFs.

The photocatalytic activity of TPCBP B-COF was investigated in TEOA (5%, basic) and sodium ascorbate (0.1 M, neutral) reaction environments and in the absence of a hole scavenger. The photocatalytic hydrogen evolution results showed that the alkaline TEOA system has the advantage of decomposition of water (no hydrogen was detected in the other media). To investigate the pH effect of the solution (from 7 to 10), the hydrogen evolution experiments were carried out at a fixed concentration of TEOA. As shown in Figure 7b, in all cases, the hydrogen evolution rate of the TPCBP B-COF photocatalyst with different pH values of TEOA was found to decrease in the following order: pH 9 > pH 10 > pH 8 > pH 7. Herein, the maximum hydrogen evolution rate was observed at pH 9, which is the highest compared to those at the more acidic and basic pH values. At the more acidic pH values, the amount of hydrogen is decreased due to the protonation of TEOA.^29^ In contrast, at the more basic value, the redox potential of H^+^/H_2_ is more negative, which is caused the low hydrogen activity.^30^

**Figure 8:**
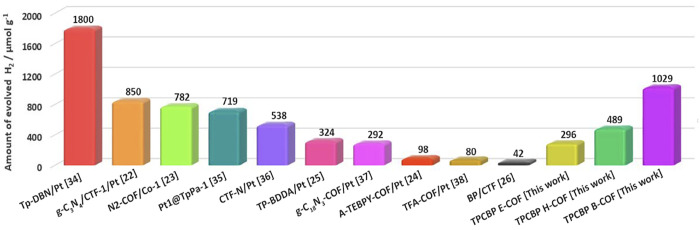
Comparison of photocatalytic H_2_ evolution performances of TPCBP X-COF (X = ethyl, butyl, and hexyl) structures with those of other published COF-based photocatalysts.

As shown in Figure 7a, highly efficient hydrogen production was achieved with TPCBP X-COF photocatalysts under the optimum conditions mentioned above. Under visible light, the performances in terms of the evolution of hydrogen from water for each photocatalyst were analyzed. According to the HER results, the produced amounts of H_2_ with TPCBP E-COF, TPCBP B-COF, and TPCBP H-COF photocatalysts were measured to be 5.165, 12.276, and 5.697 mmol g^–1^, respectively, for 8 h (Table 2). Herein, the hydrogen evolution activity of TPCBP B-COF was shown to be higher than those of TPCBP H-COF and TPCBP E-COF, which increased steadily over time (inset of Figure 7a). TPCBP E-COF is typically expected to perform better than other COF structures at producing H_2_ due to its broad light absorption and low LUMO value. In addition, upon examination of SEM and TEM images, it would be predicted to have the highest catalytic performance because it has a more porous surface area due to its crystalline structure. The HER efficiency of photocatalysts is among the key factors for the practical application of solar energy. However, as one can see from the literature on organic photovoltaics (OPVs), it is known that the exciton diffusion length is typically much shorter than the optical absorption depth.^31,32^ Because of this, we designed and investigated the effect of varying hydrocarbon chain lengths upon TPCBP X-COF photocatalysts that act as an insulating barrier for electron–hole recombination for photocatalytic HER. Herein, the appropriate bridge length of the alkyl linker in TPCBP B-COF prevented the recombination with the TPCBP electroactive structures of the bridge and caused the highest HER efficiency. It is also known that the reduction of pure domains, which serve as long-range selective charge transport channels, also leads to faster pair recombination of the electron–hole pair consisting of the same absorbed photon.^33^ Because the distance between the TPCBP electroactive structures in TPCBP E-COF is very short, it is very likely that recombination of the charges (e^–^ and hole) can occur in this structure. The TPCBP B-COF structure, which is the most ideal in terms of preventing recombination with the bridge length between the electroactive structures, was expected to have the highest efficiency. Thus, the TPCBP B-COF moieties in the quaternized bridge are the most suitable electron transfer mediator (ETM) modules, providing the most efficient electron transfer and closely collaborating with other functional modules to enhance the photocatalytic activity, according to all of the findings. Finally, the enhanced photoelectric activity of TPCBP B-COF showed that photogenerated electron–hole recombination was inhibited, and the charge transfer was more favorable. On the contrary, the flexibility of the hexyl bridge in the TPCBP H-COF structure may have decreased the photocatalytic performance by closing the holes, resulting in a decrease in the surface area as well as an increase in the band gap. Furthermore, the enhanced photocatalytic activity of TPCBP X-COF was compared with that from the literature, and our HER results are better than those for pristine COF-based photocatalysts, even with a Pt cocatalyst (Figure 8).^22−26,34−38^ Finally, the solar-to-hydrogen (STH) conversion efficiency and apparent quantum efficiency are crucial for evaluating the catalytic performance of photocatalysts. The STH conversion efficiencies of TPCBP E-COF, TPCBP B-COF, and TPCBP H-COF photocatalysts were demonstrated to be 1.26%, 2.65%, and 0.76%, respectively, which were calculated using eq S1 (see the Supporting Information). The AQEs (percent) of TPCBP B-COF were also found to be 53.72% (420 nm), 79.69% (470 nm), and 30.42% (520 nm), which are high (at 470 nm) (eq S3) for hydrogen evolution for COF-based photocatalysts, to the best of our knowledge, even in the presence of a Pt cocatalyst.^39−41^

**Table 2:** Comparison of Photocatalytic H_2_ Activities and STH Efficiencies of TPCBP X-COFs

photocatalyst	amount of H_2_ [mmol g^–1^ (8 h)^−1^]	amount of H_2_ (mmol g^–1^ h^–1^)	STH (%)
TPCBP E-COF	5.165	0.489	1.26
TPCBP B-COF	12.276	1.029	2.65
TPCBP H-COF	5.697	0.296	0.76

The recyclability of a photocatalyst is a prominent part of photocatalytic H_2_ reactions. The H_2_ evolution rate of the recycling test using TPCBP B-COF is shown in Figure 7c. After five consecutive cycles with a photocatalyst, the H_2_ evolution performance still reached 3.32 mmol g^–1^ for a 4 h illumination by maintaining a catalytic activity of 77.4%. In addition, TPCBP B-COF also exhibited long-term photocatalytic stability (HER = 25.95 mmol g^–1^ for 24 h in the inset of Figure 7a).

**Figure 9:**
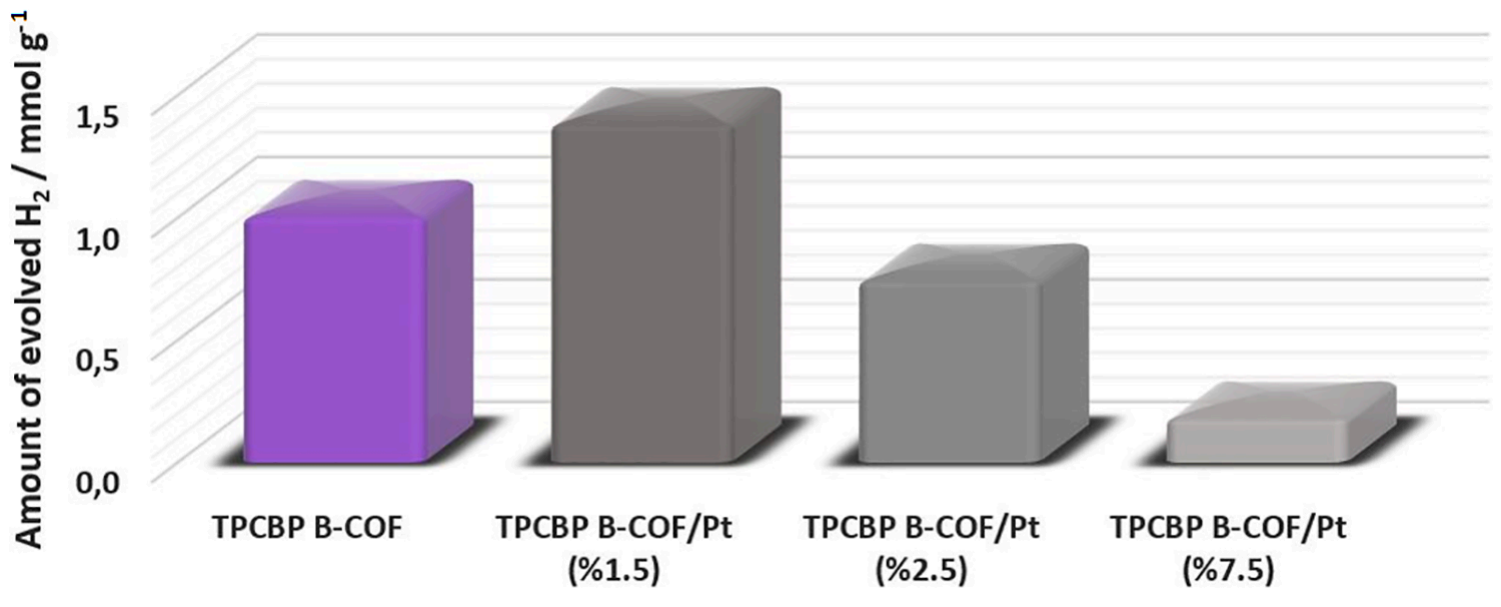
Dependence of the amount Pt on the TPCBP B-COF photocatalyst for the generation of H_2_ from water under visible light illumination.

Additionally, to investigate the Pt cocatalyst effect on TPCBP B-COF, different ratios of H_2_PtCl_6_ (1.5%, 2.5%, and 7.5%) were added to the reaction medium, affording in situ deposition of Pt. Therefore, the average amount of hydrogen of these four COFs for a 1 h illumination decreases in the following order: TPCBP B-COF/Pt (1.5%) > TPCBP B-COF > TPCBP B-COF/Pt (2.5%) > TPCBP B-COF/Pt (7.5%) (Figure 9). However, after an 8 h illumination, TPCBP B-COF with no cocatalyst (12.276 mmol g^–1^) exhibited a photocatalytic HER performance that was slightly worse than that of TPCBP B-COF with Pt (1.5%) (12.731 mmol g^–1^), which is satisfactory (Figure S10). With these results, the photocatalytic HER activity of the TPCBP B-COF/Pt photocatalyst, as a function of Pt cocatalyst loading, did not have a very crucial activity. One can conclude that pristine TPCBP B-COF can be an effective photocatalyst without using Pt, thereby considerably reducing the photocatalytic H_2_ evolution cost.

### Photocatalytic HER Mechanism of TPCBP X-COF

**Figure 10:**
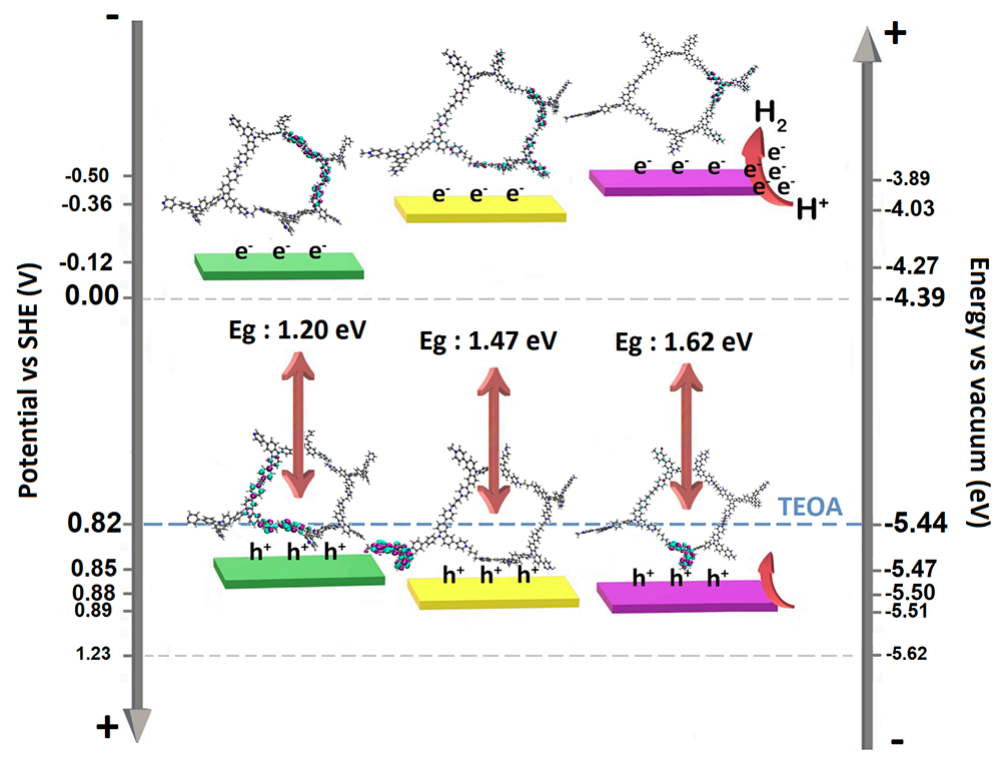
Schematic illustrations of the possible photocatalytic HER reaction mechanism over TPCBP X-COF under visible light illumination.

The photogenerated charge transfer mechanism over the TPCBP X-COFs was proposed according to the findings mentioned above (Figure 10). First, after irradiation with visible light, the excitation of TPCBP X-COF photocatalysts and the migration of light-excited electrons (e^–^) and holes (h^+^) take place by distribution of charges over pyridinium salts at the LUMO and over carbazole moieties at the HOMO, respectively. Then, photogenerated electrons at the LUMO level were transferred to the surface of TPCBP X-COFs and hydrogen generation easily occurred using protons on pyridinium salts due to the more negative LUMO band level of the photocatalysts. Charge separation at the HOMO and LUMO can be seen from the DFT calculations. On the contrary, the oxidation of donors such as triethanolamine (TEOA) is less thermodynamically demanding than that of water. It is also kinetically faster because two holes are required instead of four. Consequently, the activity of COFs for hydrogen formation is often tested using such donors rather than attempting general water splitting in the first place.^42^ Thus, the oxidation ability of TEOA was used to regenerate the photogenerated holes (h^+^) due to the HOMO levels of the TPCBP X-COF structures.

**Figure 11:**
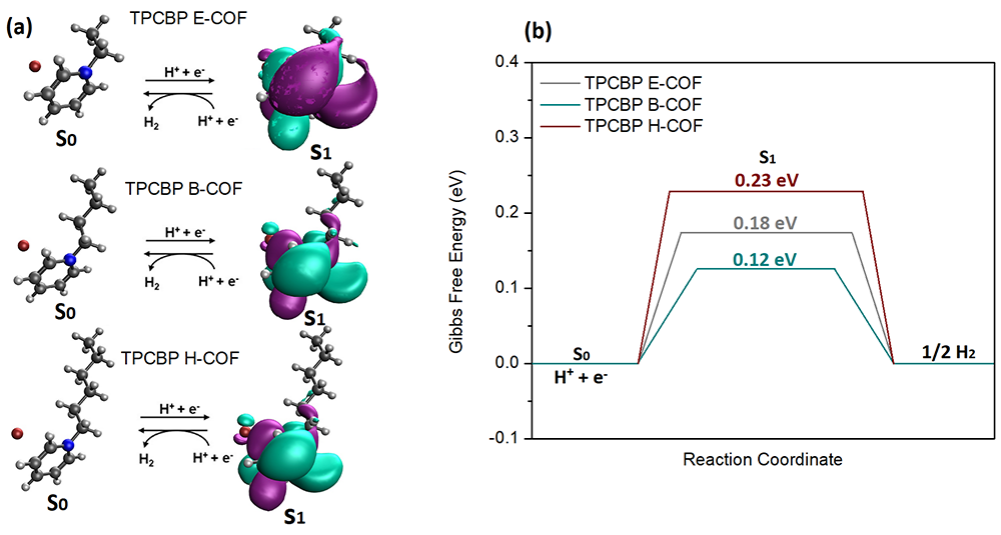
(a) Proposed H_2_ evolution reaction pathway of TPCBP X-COFs. (b) Free energy diagrams for the TPCBP X-COFs.

A three-state diagram that includes the initial state H^+^ + e^–^, intermediate-adsorbed H*, and the final product ^1^/_2_H_2_ can be used to represent the overall HER pathway. The Gibbs free energy of the molecule at the excited state has been accepted as the main descriptor of HER activity. The ideal value for the barrier energy is zero; for the well-known high-efficiency Pt catalyst, this value is as close to zero as 0.09 eV.^43,44^ Neutral and excited state DFT calculations were carried out for the pyridinium active sites, which are hydrogen carrying moieties of the TPCBP X-COFs (Figure 11). Accordingly, the energy barrier for H_2_ formation was found to be lower than that for TPCBP B-COF, which is consistent with the results of the photocatalytic tests described above.

## Conclusions

In conclusion, we described a series of highly effective metal-free photocatalytic hydrogen evolution processes by viologen-based TPCBP X-COF [X = ethyl (E), butyl (B), and hexyl (H)] structures. We observed that the different lengths of alkyl linkers in the TPCBP X-COF structures affected the optical, electrochemical, and surface properties. Accordingly, the photocatalytic hydrogen evolution performances of TPCBP X-COFs were greatly changed by the length of the alkyl chains. TPCBP B-COF has one of the highest hydrogen evolution values (1.029 mmol g^–1^ h^–1^) with the benchmark AQE of 79.69% at 470 nm among the photocatalysts, and it is crucial that this performance is demonstrated without a cocatalyst like Pt. In addition to the high activity, TPCBP B-COF also exhibited excellent stability and good reusability. The computational and experimental results suggested that the increased charge separation and the reduced energy barrier for hydrogen evolution in TPCBP X-COFs are responsible for the enhanced photocatalytic performances. Our strategy showed that the lengths of the alkyl chains in the scaffolds of COFs could dramatically change the photocatalytic hydrogen evolution performances and supply a new aspect by skeleton engineering of COFs for future solar energy conversion.

## Supporting Information Available

Synthetic procedures, instrumentation for structural characterization, FT-IR spectra, thermal analysis, powder XRD analysis, electrochemical and optical properties, geometric optimization calculations, SEM-EDX analysis, photocatalytic hydrogen evolution experiments, calculation of AQE, effect of different Pt ratios on the process, and the photocatalyst recycling procedure (PDF)
