# Supplementary material for: Viologen-Based Covalent Organic Frameworks toward Metal-Free Highly Efficient Photocatalytic Hydrogen Evolution
Source: ACS Appl Mater Interfaces. 2023 Apr 5;15(15):18836–44. doi: 10.1021/acsami.2c23233 (PMC10119857; doi:10.1021/acsami.2c23233)
Supplement: Supplementary file 1 — am2c23233_si_001.pdf [file am2c23233_si_001.pdf]

Supporting Information For

Viologen-Based Covalent Organic Frameworks Toward Metal-Free Highly Efficient Photocatalytic Hydrogen Evolution

Sinem Altınışık[a,b], Gizem Yanalak[c], İmren Hatay Patır[d] and Sermet Koyuncu[a,b*]*

[a] Canakkale Onsekiz Mart University, Department of Chemical Engineering, 17100, Çanakkale, Türkiye.

[b] Canakkale Onsekiz Mart University, Department of Energy Recourses and Management, 17100, Çanakkale, Türkiye.

[c] Selcuk University, Department of Biochemistry, 42130 Konya, Türkiye.

[d] Selcuk University, Department of Biotechnology, 42130, Konya, Türkiye.

*Corresponding authors: imrenhatay@gmail.com (İ.H.P) and skoyuncu@comu.edu.tr (S.K.)

Table of Contents

**Materials and Synthetic Procedures**	**S-2**
**Instrumentation for Structural Characterization**	**S-3**
**FT-IR Spectra**	**S-4**
**Thermal Analysis**	**S-5**
**Electrochemical and Optical Properties**	**S-6**
**Geometric Optimization Calculations**	**S-7**
**Powder X-Ray Diffraction Analysis**	**S-8**
**SEM-EDX Analysis**	**S-8**
**Photocatalytic Hydrogen Evolution Experiments**	**S-9**
**The Apparent Quantum Efficiency (AQE) Calculation**	**S-9**
**Effect of Different Pt Ratios on Process**	**S-11**
**Photocatalyst Recycling Procedure**	**S-11**
**References**	**S-12**

A. Materials and Synthetic Procedures

Materials

All chemicals were purchased from Sigma-Aldrich and Merck as a commercial supplier. 4,4′-di(carbazol-9-yl)-biphenyl (CBP)[1] (1) and 4,4'-bis(3,6-dibromo-9H-carbazo-9-yl)-1,1'-biphenyl (TBCBP)[2] (2) were synthesized with slightly modifications from previously reported procedures. The synthetic procedure of TPCBP (3) and TPCBP X-COF (4) structures are given in Figure S1.

Synthetic Procedures

The 4,4'-bis(3,6-dipyridine-9H-carbazo-9-yl)-1,1'-biphenyl (TPCBP) (3) was synthesized in one step using TBCBP (2) and Pyridine-4-boronic acid pinacol ester via Suzuki reaction. In a 100 mL dried Schlenk flask, TBCBP (2) (1.00 g, 1.24 mmol) and Pyridine-4-boronic acid pinacol ester (1.29 g, 6.63 mmol) were dissolved in 40 mL Toluene and 5 mL of 2.0 M K_2CO_3 for 15 minutes by purging with argon atmosphere. Tetrakis(triphenylphosphine)-palladium(0) ($Pd(PPh_3)_4$) (0.40 g, 0.35 mmol) as catalyst was added to the reaction mixture at about 80°C. For 24 hours, the mixture was stirred at 110 °C in an argon atmosphere. The reaction mixture was cooled to room temperature after 24 hours and then poured into 250 ml of ethanol. After the solvent evaporated, the precipitated salts were filtrated, and the material crystallized in ethanol. TPCBP was produced after the pure gray color product dried at 60 °C under vacuum for 12 hours. And then, TPCBP (1eq) and a series of dibromo compounds (2.1 eq) (1,2-dibromoethane, 1,4-dibromobutane, and 1,6-dibromohexane) were added to the round bottom flasks in 20 mL of DMF at 180 °C. Initially the solution was turned into yellow and then precipitated in the reaction mixture. After the 48h, the final products were separated by filtration. Finally, the TPCBP X-COF structures were dried for 12 hours at 80 °C in a vacuum.

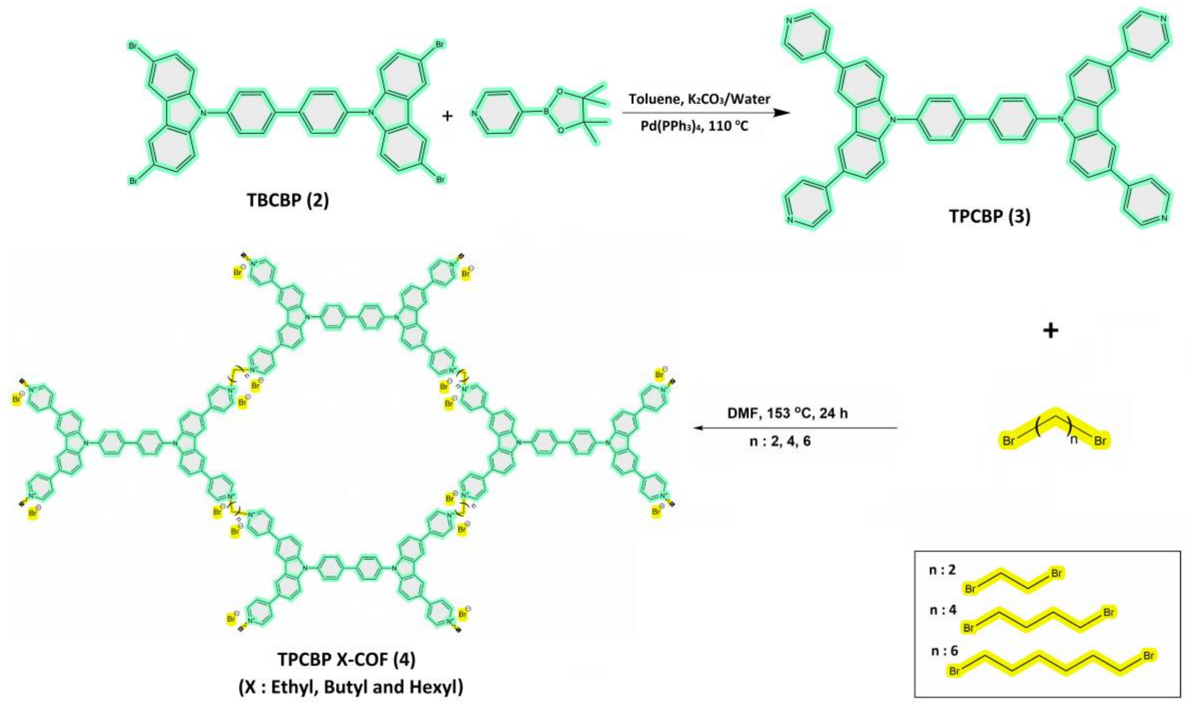

Figure S1. Synthesis of the TPCBP (3) and TPCBP X-COF (X: ethyl (E), butyl (B), and hexyl (H)).

B. Instrumentation for Structural Characterization

Fourier-transform infrared spectroscopy (FT-IR) spectra were recorded on Cary 630 FTIR Spectrometer (Agilent Technologies) an ATR system (4000-650 cm^{-1}). Thermogravimetric analyses (TGA) were performed using a Perkin Elmer TGA 8000 under a nitrogen atmosphere at 10 °C/min temperature. Powder X-ray diffraction (XRD) profiles were collected on a Panalytical Empyrean. Using a CH Instruments 617D potentiostat/galvanostat system, electrochemical analyses were carried out in argon atmosphere. In this system, the working electrode (WE) is a Pt disc (0.02 cm^2), the counter electrode (CE) is a Pt wire, and the pseudo-reference electrode (PRE) is an Ag wire. Electrolyte solution containing 0.1 M TBAPF6 in acetonitrile (ACN) as supporting electrodes was used. The electrochemical HOMO-LUMO band gap of TPCBP X-COFs calculated from the oxidation-reduction onset potentials was calibrated against the ferrocene redox couple using the equation $E_{HOMO} = -e (E_{ox\text{-}ons} - E_{Fc}) + (-4.8\ eV)$ and $E_{LUMO} = -e (E_{red\text{-}ons} - E_{Fc}) + (-4.8\ eV)$.[3] This values vs Ag/AgCl was converted to

against Standard Hydrogen Electrode (SHE) by adding 0.230 V.[4] Analytic Jena Speedcord S-600 diode-array spectrophotometer was used to measure UV-Vis absorption spectra. The optical band gaps (Eg') of the COF structures were calculated from the absorption edges (λ_{onset}) using the equation Eg' = 1241/λonset.[5] Theoretical calculations were performed using Gaussian 09, and visualization was performed using GaussView 5.0. Geometry optimization of TPCBP X-COFs were performed in the 6-31+G(d,p) basis set functional integrated with B3LYP in DFT method. The TEM images were obtained using an 80 kV operating voltage JEOL JEM 1400 Plus TEM device (Tokyo, Japan). 10 µL of ethanol solution was deposited on a carbon supported copper 300 mesh grid after sonication for 1 min and allowed to dry at room temperature for 1 hour prior to analysis. Additionally, Scanning Electron Microscopy was used to image the surface morphologies (SEM, JEOL JSM-7100-F). To provide elemental characterization and quantitative compositional data, an Energy Dispersive X-Ray Analyzer (EDX) integrated with SEM was also used.

C. FT-IR Spectra

TPCBP (Yield: 80%), FT-IR wavenumber (cm^{-1}) = 3054, 3033, 1628,1590, 1544, 1497, 1470, 1454, 1408, 1361, 1340, 1320, 1280, 1220, 1168, 1100, 1029, 994, 798, 743, 667.

TPCBP E-COF (Yield: 72%), FT-IR wavenumber (cm^{-1}) = 3370, 3038, 1620, 1588, 1497, 1473, 1359, 1320, 1285, 1214, 1190, 1147, 1002, 803, 713, 656.

TPCBP B-COF (Yield: 95%), FT-IR wavenumber (cm^{-1}) = 3376, 3033, 2929, 1620, 1587, 1498, 1479, 1359, 1321, 1282, 1220, 1171, 1144, 1002, 804, 722, 659.

TPCBP H-COF (Yield: 87%), FT-IR wavenumber (cm^{-1}) = 3378, 3033, 2930, 2856, 1620, 1590, 1498, 1476, 1359, 1320, 1285, 1220, 1173, 1147, 1002, 806, 722, 664.

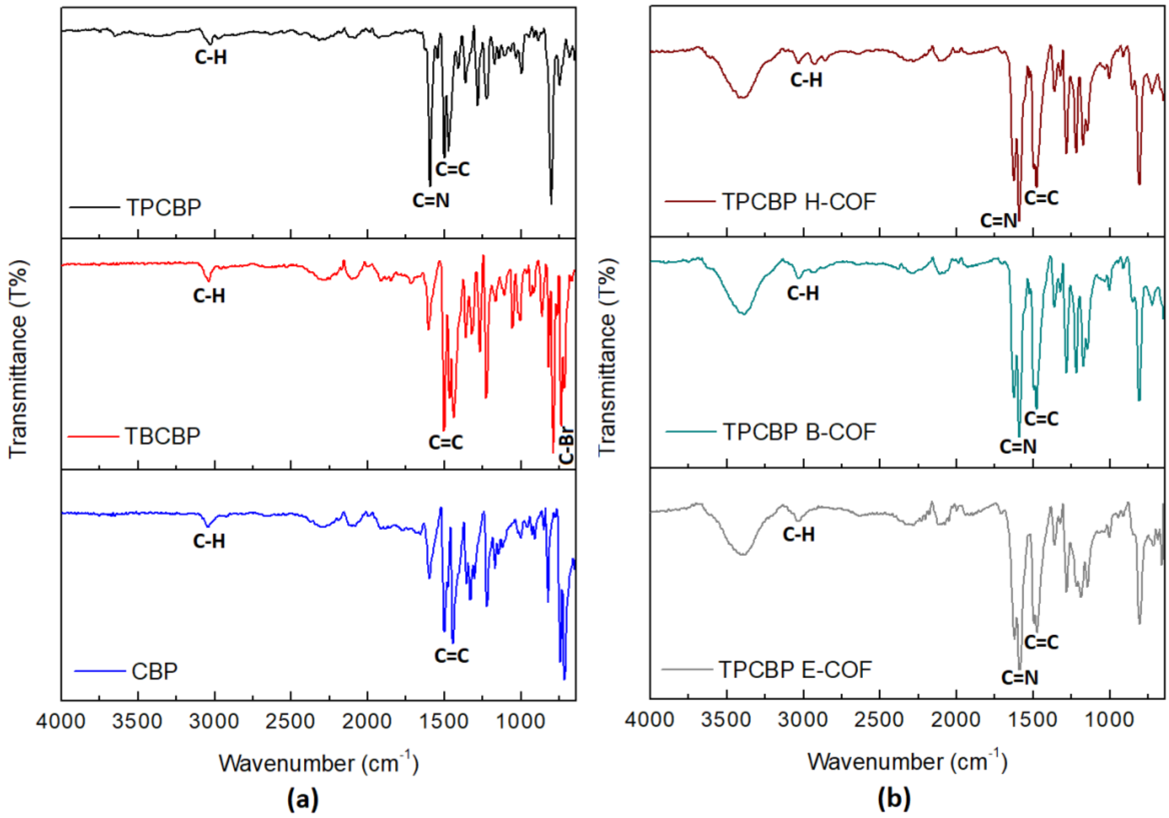

Figure S2. FT-IR spectra of initial compounds and COF structures (TPCBP X-COF).

D. Thermal Analysis

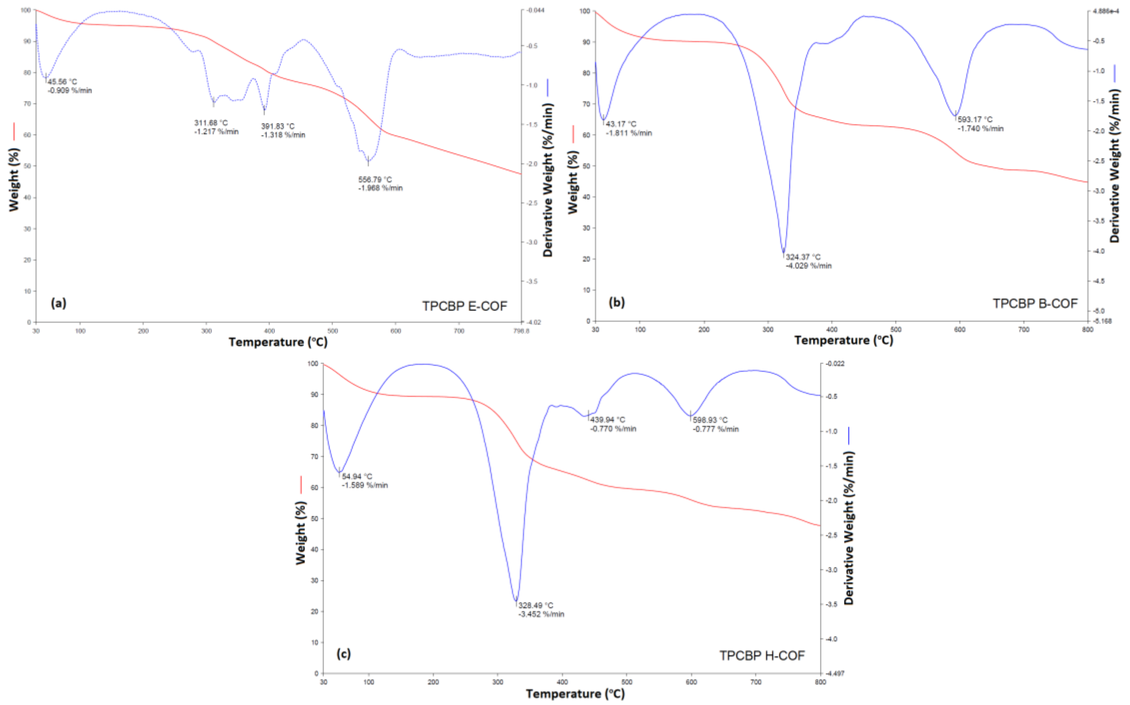

Figure S3. Thermogravimetric analysis (TGA) curves for TPCBP E-COF (a), TPCBP B-COF (b) and TPCBP H-COF (c).

E. Electrochemical and Optical Properties

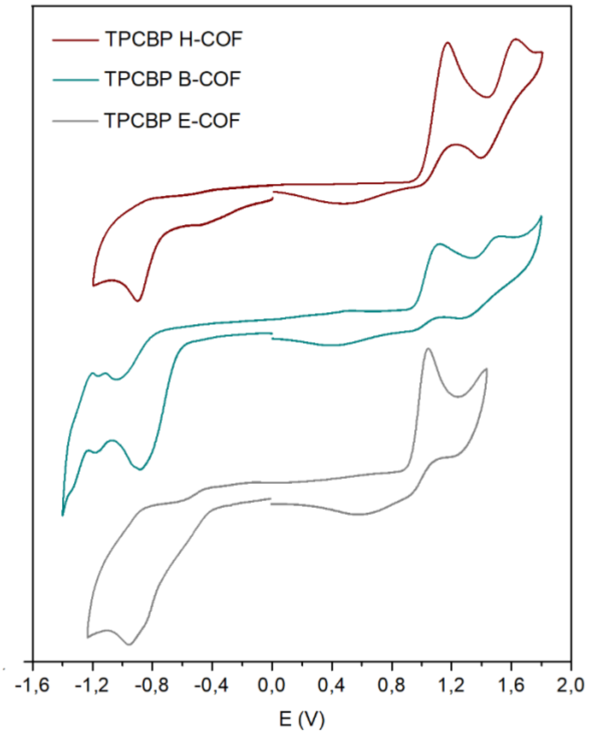

Figure S4. CV curves of TPCBP X-COF in 0.1 M TBAPF$_6$/ACN electrolyte solution at scan rate of 100 mV/s, Ag wire.

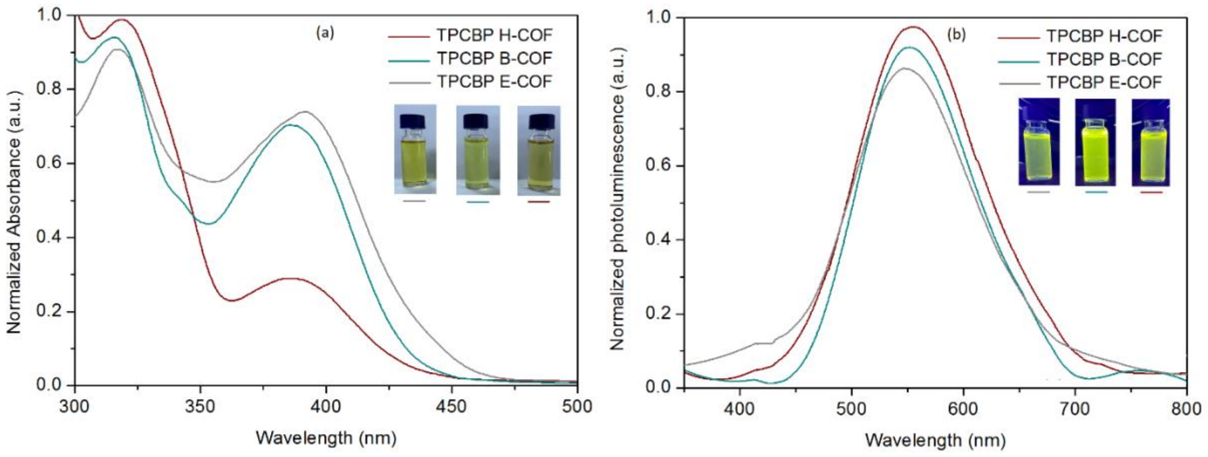

Figure S5. (a) UV-Vis absorption spectra of TPCBP X-COF in DMF (b) Photoluminescence spectra of TPCBP X-COF in DMF.

Table S1. Electrochemical and optical values of TPCBP X-COFs (Potential determined versus SHE and Ag/AgCl).

Molecule	Oxidation Onset Potential (V) vs Ag/AgCl	Oxidation Onset Potential (V) vs SHE	Reduction Onset Potential (V) vs Ag/AgCl	Reduction Onset Potential (V) vs SHE	*HOMO (eV) vs Ag/AgCl	**HOMO (eV) vs SHE	*LUMO (eV) vs Ag/AgCl	**LUMO (eV) vs SHE	Eg (eV)	Eg' (eV)
TPCBP E-COF	0.85	1.08	-0.35	-0.12	-5.24	-5.47	-4.04	-4.27	1.20	2.58
TPCBP B-COF	0.88	1.11	-0.59	-0.36	-5.27	-5.50	-3.80	-4.03	1.47	2.63
TPCBP H-COF	0.89	1.12	-0.73	-0.50	-5.28	-5.51	-3.66	-3.89	1.62	2.73

*The electrochemical HOMO-LUMO band gap of TPCBP X-COFs calculated from the oxidation-reduction onset potentials was calibrated against the ferrocene redox couple using the equation $E_{HOMO} = -e(E_{ox-ons} - E_{Fc}) + (-4.8\ eV)$ and $E_{LUMO} = -e(E_{red-ons} - E_{Fc}) + (-4.8\ eV)$ (E_{Fc} is taken as 0.41). **This values vs Ag/AgCl was converted to against Standard Hydrogen Electrode (SHE) by adding 0.230 V.

F. Geometric Optimization Calculations

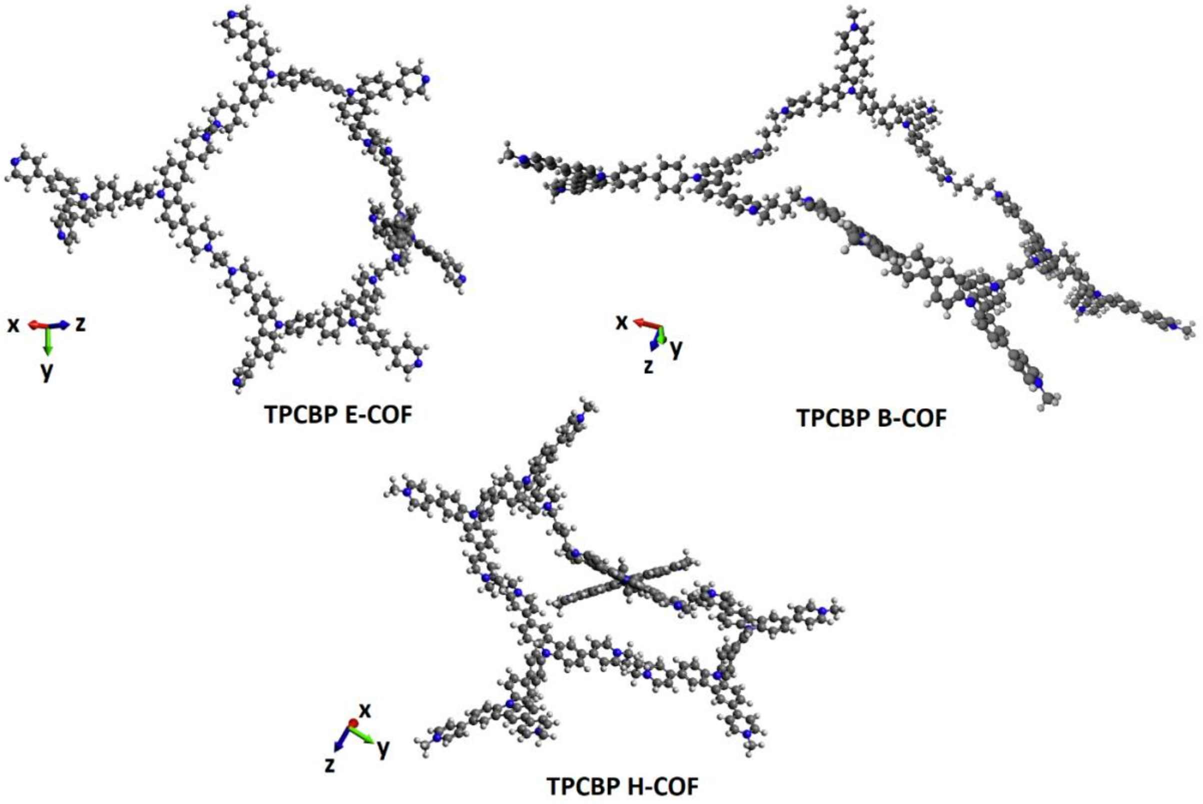

Figure S6. After geometric optimization, images of TPCBP X-COF structures from a variety of perspectives.

G. Powder X-Ray Diffraction Analysis

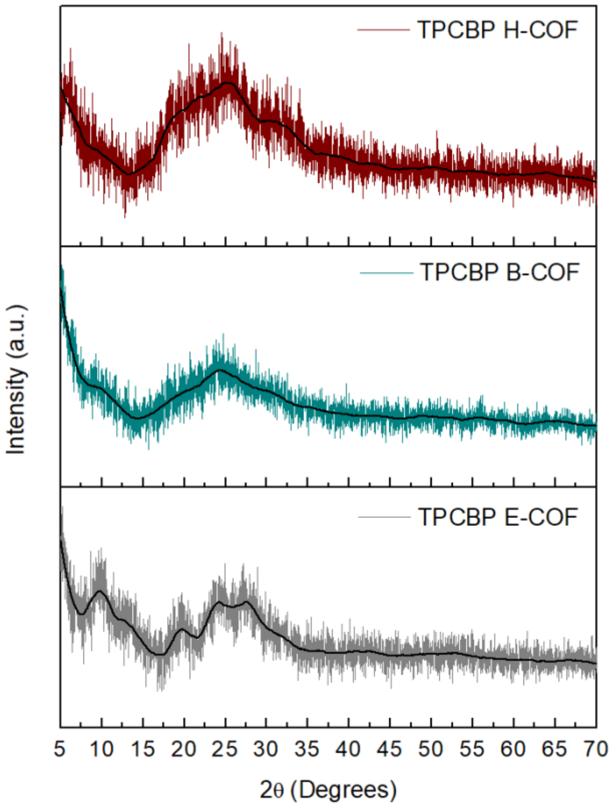

Figure S7. XRD patterns of TPCBP X-COF powders.

H. SEM-EDX Analysis

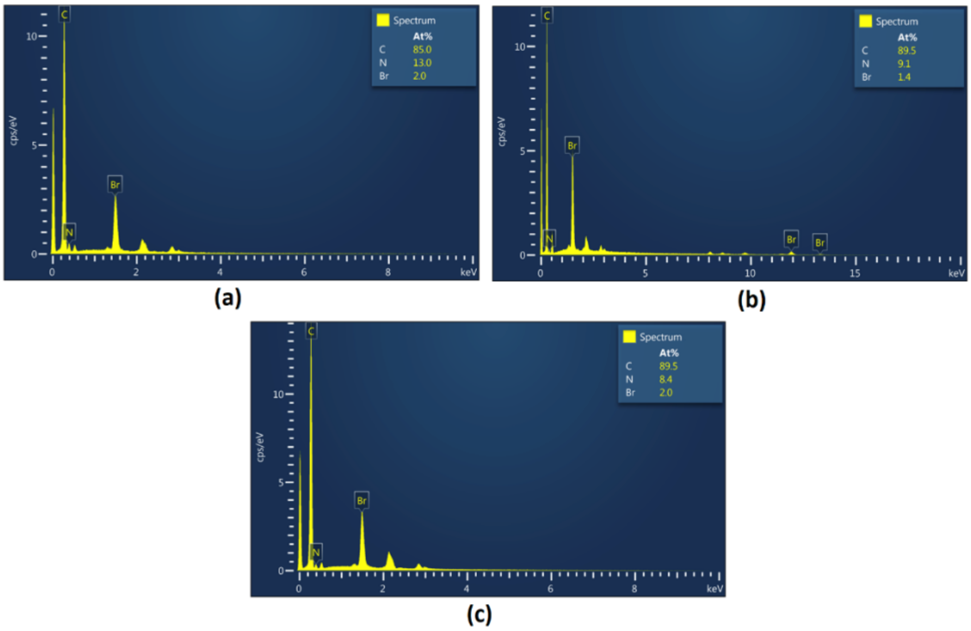

Figure S8. Energy-Dispersive X-ray spectroscopy (EDX) pattern of TPCBP X-COFs dispersed in ethanol solution (a-c) (X: ethyl, butyl, and hexyl), respectively.

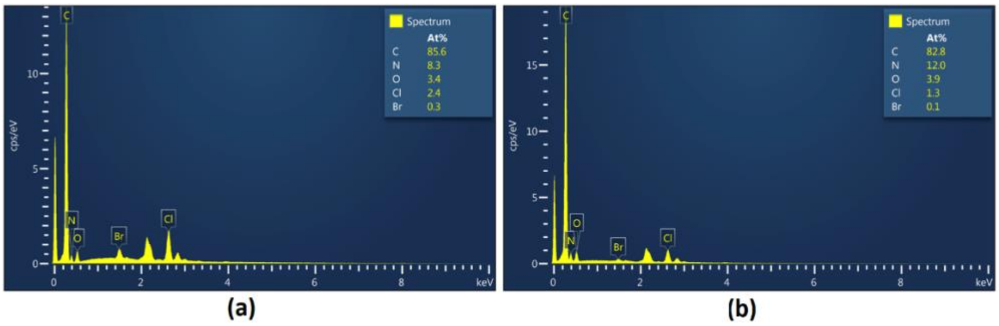

Figure S9. Energy-Dispersive X-ray spectroscopy (EDX) pattern of TPCBP B-COF under 8 h visible light illumination (a) and after photocatalytic stability test (b), respectively.

I. Photocatalytic Hydrogen Evolution Experiments

To assess the photocatalytic H_2 evolution of as-fabricated TPCBP X-COF, the experiments were performed under visible-light illumination by using 300 W Xe lamp with a cut-off filter (λ>420 nm). In a typical procedure, the photocatalyst and TEOA electron mediator solution with different pH values were added into the reaction vessel in a glovebox atmosphere. Then, the suspension was sealed with a rubber septum and sonicated to obtain a homogeneous solution. The above photocatalytic reaction system was stirred under visible-light conditions. The H_2 evolved in the headspace of the vessel was determined by gas chromatography (Shimadzu GC-2100Plus) equipped with a thermal conductivity detector (TCD). In addition, to determine the effect of Pt co-catalyst on TPCBP X-COF, chloroplatinic acid (H_2PtCl_6) was used to as a precursor for its in-situ reduction to metallic platinum.[6]

The solar to hydrogen (STH) conversion efficiency is calculated according to the following formula:

$$STH\ (\%) = \left[\frac{R_{H_2}\ x\ \Delta G^{o}}{P\ x\ A}\right]_{AM\ 1.5G} x\ 100 \qquad (S1)$$

where R_{H2}, $\Delta G°$, P, and S denote the generation of H_2 (mmol s^{-1}) in the <u>photocatalytic splitting</u> of water, <u>standard Gibbs energy</u> for generating one mole of H_2 (J mol^{-1}), the intensity of sunlight (mW cm^{-2}) and irradiation area (cm^2), respectively.

J. The Apparent Quantum Efficiency (AQE) Calculation

The apparent quantum efficiencies (AQE) of TPCBP X-COF (X: ethyl, butyl, and hexyl) for HER evolution were measured using the 420 nm, 470 nm, and 520 ± 20 nm band-pass filter. The AQE of each catalyst was defined according to the following equations.

$$\text{AQE [\%]} = \frac{\text{Number of reacted electron}}{\text{Number of incident photons}} \text{x} 100 \quad \text{(S2)}$$

Finally, the AQE yield was calculated using the following equation to make a clearer explanation,

$$\text{A.Q.Y (\%)} = [(2 \times M \times N_A \times h \times c) / (P \times S \times t \times \lambda_{inc})] \times 100 \quad \text{(S3)}$$

where, M = Amount of hydrogen produced (mol), N_A = Avogadro number (6.022×10^{23} 1/mol), c = Speed of light (3×10^8 m/s), h = Planck′s constant (6.626×10^{-34} J/s), P = Power density of the incident monochromatic light (mW/cm^2), S = Irradiated area to produce M (mol) of hydrogen (cm^2), t = Irradiated time (1 h = 3600 s), λ_{inc} = wavelength of the incident monochromatic light (nm).[7]

According to the above-mentioned equation, the AQYs were calculated based on the amounts of H_2 for TPCBP B-COF photocatalyst under monochromatic light irradiation in one hour.

Table S2. Apparent quantum yields of TPCBP B-COF sample.

λ_{inc} (nm)	Irradiation area (cm^2)	Power density (μW/cm^2)	Amount of H_2 production (μmol/h)	AQY (%)
420	25.52	1000	86.58	**53.72**
470		1600	229.96	**79.69**
520		1800	109.26	**30.42**

K. Effect of Different Pt Ratios on Process

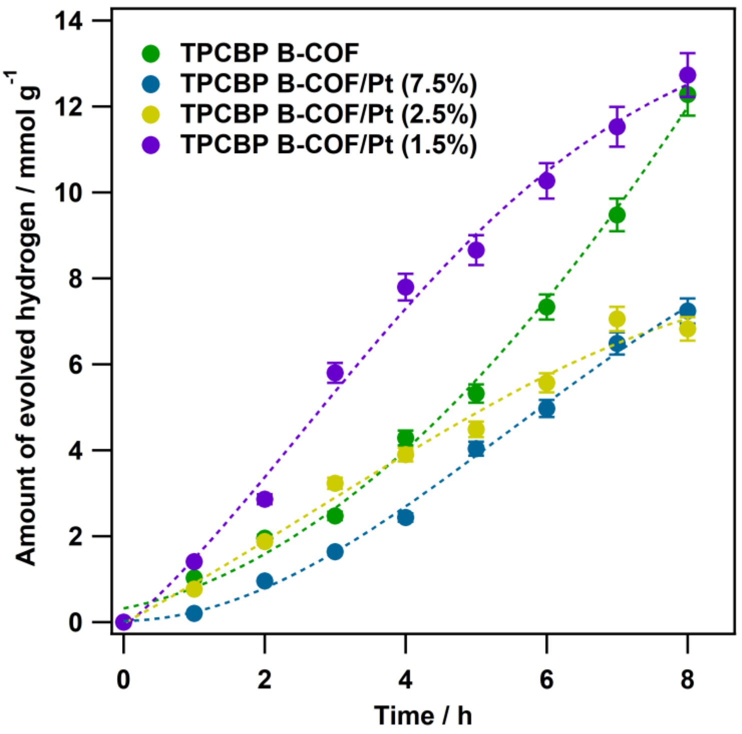

Figure S10. The photocatalytic hydrogen evolution test of the different ratio of Pt loaded on TPCBP B-COF and bare TPCBP B-COF with the TEOA sacrificial reagent under visible light illumination (λ > 420 nm).

L. Photocatalyst Recycling Procedure

For the recyclability tests of TPCBP X-COF (X: ethyl, butyl, and hexyl), the reaction mixture was taken and centrifugated for 10 min. at 4500 rpm for 2 times. The precipitated photocatalyst was harvested, then rinsed with distilled water and dried at room temperature. Eventually, the procedure for the photocatalytic H_2 evolution was repeated for the photocatalyst as described above.

M. References

(1) Koyuncu, S.; Gultekin, B.; Zafer, C.; Bilgili, H.; Can, M.; Demic, S.; Kaya, İ.; Icli, S. Electrochemical and optical properties of biphenyl bridged-dicarbazole oligomer films: Electropolymerization and electrochromism. *Electrochimica Acta* **2009**, *54* (24), 5694-5702.
(2) Stoeck, U.; Krause, S.; Bon, V.; Senkovska, I.; Kaskel, S. A highly porous metal–organic framework, constructed from a cuboctahedral super-molecular building block, with exceptionally high methane uptake. *Chemical Communications* **2012**, *48* (88), 10841-10843.
(3) Heinze, J.; Frontana-Uribe, B. A.; Ludwigs, S. Electrochemistry of Conducting Polymers—Persistent Models and New Concepts. *Chemical Reviews* **2010**, *110* (8), 4724-4771.
(4) Vega, C. A.; Delgado, S. Standard potential of the silver/silver chloride electrode in 50 wt. % 2-methoxyethanol-water solvent from 5 to 45.degree.C. *Journal of Chemical & Engineering Data* **1986**, *31* (1), 74-76.
(5) Patil, A. O.; Heeger, A. J.; Wudl, F. Optical properties of conducting polymers. *Chemical Reviews* **1988**, *88* (1), 183-200.
(6) Tossi, C.; Hällström, L.; Selin, J.; Vaelma, M.; See, E.; Lahtinen, J.; Tittonen, I. Size- and density-controlled photodeposition of metallic platinum nanoparticles on titanium dioxide for photocatalytic applications. *Journal of Materials Chemistry A* **2019**, *7* (24), 14519-14525.
(7) Yu, Y. G.; Chen, G.; Hao, L. X.; Zhou, Y. S.; Wang, Y.; Pei, J.; Sun, J. X.; Han, Z. H. Doping La into the depletion layer of the Cd(0.6)Zn(0.4)S photocatalyst for efficient H(2) evolution. *Chem Commun (Camb)* **2013**, *49* (86), 10142-10144.
